# Heart transplantation of patients with ventricular assist devices: impact of normothermic ex-vivo preservation using organ care system compared with cold storage

**DOI:** 10.1186/s13019-020-01367-w

**Published:** 2020-10-27

**Authors:** Rymbay Kaliyev, Timur Lesbekov, Serik Bekbossynov, Zhuldyz Nurmykhametova, Makhabbat Bekbossynova, Svetlana Novikova, Assel Medressova, Nurlan Smagulov, Linar Faizov, Robertas Samalavicius, Yuriy Pya

**Affiliations:** 1 National Research Center for Cardiac Surgery, Nur-Sultan, Kazakhstan; 2 Vilnius University Hospital Santariskiu Klinikos, Vilnius, Lithuania

**Keywords:** Heart transplantation, Ex vivo organ preservation, Mechanical circulatory support, High-risk recipients

## Abstract

**Background:**

Organ Care System (OCS) minimizes the cold ischemic time and allows for optimization of logistics and meticulous recipient preparation. Impact of normothermic ex-vivo preservation using OCS compared with cold storage (CS) for prolonged heart preservation especially beneficial for high-risk recipients bridged to transplantation with Mechanical Circulatory Support (MCS).

**Methods:**

Between 2012 and 2018, we performed a retrospective single-center review of prospectively collected data. All patients who underwent heart transplantation with MCS using the OCS Heart (*n* = 25) versus standard cold storage (*n* = 10) were included in this study.

**Results:**

During this period, 353 patients were implanted with left ventricular assisted device (LVAD) and 35 (10%) were bridged to heart transplantation. There was no significant difference in donor and recipient characteristics and risk factors. The Index for Mortality Prediction after Cardiac Transplantation (IMPACT) score was a trend towards higher estimated risk of death at 1y in the OCS group (14.2 vs. 10.8% *p* = 0.083). Mean total ischemic time during preservation was statistically significantly longer in CS vs OCS group (210 (23) Vs 74.6 (13) min *p* = 0.001). Median ex vivo normothermic heart perfusion time in OCS was 348.4(132; 955) min. There was significant difference in total out of body time between OCS group 423(67) Vs CS group 210(23) min *p* = 0.002). All patients were alive on the 30th days post implant in CS groups and 96% in OCS group *(p* = *0.5)*.

**Conclusion:**

Normothermic ex-vivo preservation of the allograft during transportation with the organ care system might be beneficial for long-time out of body organ preservation in comparison of cold storage especially for recipients on mechanical circulatory support.

## Background

Despite improvements of mechanical circulatory support in recent years, heart transplantation remains the approach most likely to improve survival and quality of life in patients with end-stage heart failure. Success in heart transplant depends on the quality of the donor heart, procurement, preservation and storage of the graft, the complexity of the operation and duration of graft ischemia. Some determinants of successful transplant outcomes are difficult or even impossible to modify, such as the recipient co-morbidities or the quality of the donor heart. [1, 2]. Survival after heart transplantation has improved but primary graft dysfunction (PGD) remains a significant problem and cause of early mortality [3]. Under conventional conditions of donor organ preservation, i.e., cardioplegic arrest and cold storage, prolonged cold ischemia time is by far the greatest risk factor for primary allograft dysfunction and death [4]. The individual risk–benefit ratio is further affected by the ever-increasing complexity of today’s recipients, such as the presence of LVAD with severe pulmonary hypertension. In particular, transplantation in patients with LVADs is challenging, and the concept of LVAD bridging on outcomes after transplantation has been controversial. Some experienced centers have comparable post transplantation results in this group of patients [5, 6]. However, the international registries continue to identify it as a risk factor for increased mortality [7, 8]. Ex vivo normothermic preservation, using OCS minimizes the cold ischemic time and allows for optimization of logistics and meticulous recipient preparation. In 2011, we initiated the heart failure program in our country and up to now 353 patients underwent implantation of long-term mechanical circulatory support. In the 2012, we initiated heart transplantation program in Kazakhstan, and to date 80 heart transplanted. Our center is sole transplant center in our country, and donor hearts are often retrieved from distant regions to be transplanted at our center.

In this article, we report a single-center experience of impact of normothermic ex-vivo preservation using organ care system compared with Cold storage for prolonged heart preservation especially beneficial for high-risk recipients bridged to transplantation with Mechanical Circulatory Support.

## Methods

### The Heart OCS

The heart OCS (Transmedics Inc, Boston, MA) is composed of an organ perfusion module with disposable and non disposable parts and a compact wireless monitor. The monitor displays indicated (online time) organ measurements, such as aortic pressure, coronary flow, blood temperature, and heart rate. The heart is perfused in the resting mode. Warm oxygenated blood is pumped into the aorta, thereby perfusing the coronary arteries, and deoxygenated blood enters the right atrium through the coronary sinus and passes through the tricuspid valve to the right ventricle. The blood is then ejected through the pulmonary artery to the blood oxygenator and is returned to the reservoir.

### Procedures

After acceptance of donor heart based on clinical information, our team performed a detailed allograft assessment at the time of donation (transesophageal echocardiography, cardiac output studies using a pulmonary artery catheter, direct evaluation of the coronary arteries, and measurement of left and right atrial pressures). Before aortic cross clamping, the right atrial appendage was cannulated using a 34F venous cannula, thereby allowing approximately 1.5 L of donor blood to be collected to prime the OCS module. After the donor was heparinized (300 IU/kg), the donor blood was collected prior to antegrade cardioplegia and prior to cross clamping of the aorta. In blood collection bag was added heparin (10,000 IU) and this was used to prime the perfusion module. Portion of the normothermic blood (500–750 mL) was collected retrogradely for initial dose of blood cardioplegia. The aorta and pulmonary artery of the donor heart were cannulated and heart connected to the OCS with the posterior aspect facing upward and the left atrium and aorta toward the heart chamber. In the OCS, oxygenated blood was pumped into the aorta, perfusing the coronary arteries. The coronary sinus flow then passes through the tricuspid valve (as both the superior and inferior vena cavae are sutured closed) and is ejected by the right ventricle into a pulmonary artery catheter and returned to the blood reservoir. Then, the heart is reanimated to normal sinus rhythm. The pump flow and solution flow rates of the OCS were adjusted to maintain the mean aortic pressure between 60 and 90 mmHg and coronary blood flow between 650 mL/min and 850 mL/min. According to standard protocol, samples were taken in the OCS before the donor heart was connected to the OCS. These included donor lactate (CG4 + , within 30 min of blood collection), baseline OCS lactate and chemistries (CG8 + , during priming). Periodic arterial chemistry samples were taken during OCS time (approximately every 20–30 min). Samples were collected from the arterial and venous sampling port of OCS. The samples were analyzed with a handheld lactate analyzer (i-STAT, Abbott Diagnostics, East Windsor, NJ, USA). Upon arrival at recipient center, the donor heart was arrested with approximately one liter of normothermic blood cardioplegia before transplanting. The graft was conditioned with Levosimendan 45 µg/kg (using body weight of donor) while in the OCS and hemofiltration with a blood flow of 200–300 ml/h was applied in the OCS in order to protect and improve donor heart function.

For the standard cold storage group, the donor heart was arrested with the standard heart preservation solution (4^0^C Custodiol). Transplantation and preoperative care proceeded according to the standard procedures of our center in both groups.

### Study Design and Participants

From 2011, when initiated the heart failure program 353 patients were implanted with ventricular assist devices to date and 35 (10%) of them transplanted [9]. Between 2012 and 2018, we performed a retrospective single-center review of prospectively collected data. All patients who underwent heart transplantation with MCS using the OCS Heart (*n* = 25) versus standard cold storage were included in this study. Eligible recipients were at least 18 years of age and had to be on the heart-transplant waiting list. The study received approval through the responsible ethics committee at our institution and all patients provided written informed consent to be part of this study and to allow their data to be used for the analysis. Endpoints included 30-day survival, heart preservation time (ischemic time, OCS perfusion time, out of body time), duration and level of inotropic support, ITU stay-day, Mechanical Circulatory Support after heart transplantation, adverse cardiac events.

### Statistical analysis

Results were expressed as mean and standard deviation or median and interquartile range (continuous variables), and counts with percentages (categorical variables). Where possible, a two-sample independent t-Test was used to compare the means. Outcome measures used were 30-day survival. Statistical analyses were performed using STATA version 12 (Stata Corp, Texas, US).

## Results

### Donor and Recipient population

The donor and recipient characteristics and risk factors are presented in Table 1. There was a trend slightly higher donor age on the OCS group vs CS (41.3 ± 9.3 Vs 38.3 ± 11.5 yo; *p* = 0.2), with 92% vs 70% male donors. The gender mismatches among the donor/recipients profile in OCS group 3 male donor to 3 female recipient, and in SC group 3 female donor to 3 male recipient. Nineteen donors (76%) vs six (60%) died of spontaneous intracranial hemorrhage, 6 (24%) vs 3 (30%) died of cerebrovascular accidence in OCS vs CS group respectively, and 1(10%) patient died of trauma in CS group.

**Table 1:** The donor and recipient characteristics and risk factors

	OCS (*n* = 25)	CS (*n* = 10)	*P* value
Donor characteristics			
Age (years)	41.3 ± 9.3	38.3 ± 11.5	0.2
Male, n (%)	23(92)	7(70)	0.9
Cause of death, n (%)			
Intracranial hemorrhage	19 (76)	6 (60)	0.9
Cerebrovascular accident	6 (24)	3 (30)	0.9
Trauma		1 (10)	
Median LVEF (range)	58(52–63)	60(54–65)	
Recipient characteristics			
Age (years)	38.6 ± 11.9	43.6 ± 12.6	0.2
Male, n (%)	20(80)	10(100)	0.7
NICM n (%),	16(64)	7(70)	0.9
Median previous sternotomies rate	2(1;5)	1(1;3)	0.1
PVR > 4WU	4(16%)	4(40%)	0.6
Mechanical Circulatory Support			
LVAD, n (%)	20(80)	10(100)	
ECMO, n (%)	2(8)		
CARMAT, n (%)	3(12)		

Data are expressed as mean ± standard deviation, unless otherwise noted

*NICM* non-ischaemic cardiomyopathy, *LVEF* left ventricular ejection fraction, *LVAD* left ventricular assist device, *ECMO* Extracorporeal Membrane Oxygenator, *CARMAT* total artificial heart, *PVR* pulmonary vascular resistance, *WU* Wood unit

There was no significant difference in recipient age in OCS and CS group (38.6 ± 11.9 Vs 43.6 ± 12.6 yo; *p* = 0.2) and 80% (*n* = 20) vs 100% (*n* = 10) were male, respectively. All patients had advanced heart failure (64% vs 70% NICM) in OCS vs CS group. The IMPACT score was a trend towards higher estimated risk of death at 1y in the OCS group (14.2 vs. 10.8% *p* = 0.083).

In the OCS group 20 recipients was on LVAD support (HeartWare-3, HeartMate II- 10, HeartMate 3- 4, HeartMate 3 + ECMO- 1, HeartMate 3 + RVAD-1, RVAD + LVAD (short term biVAD Levitronix)-1) and ECMO-2, total artificial heart (CARMAT)-3 compared in the CS group 10 recipients on LVAD support (HeartWare-2, HeartWare + RVAD-1, HeartMate II-4, HeartMate 3 -2, HeartMate 3 + RVAD (Levitronix)-1). Of the 20 recipients who received LVAD support preoperatively, six versus two patients had an ongoing severe pump pocket infection at the time of transplantation in OCS and CS, respectively. Two patients in OCS group vs one patient in CS group were on inotropic support in addition to MCS preoperatively milrinone 0.1 vs 0.15 mcg/kg/min, dobutamine 7 vs 6 mcg/kg/min, respectively.

### OCS assessment

Mean (SD) total ischemic time during preservation was statistically significantly longer in CS group in comparison with OCS group 210 (23) Vs 74.6 (13) min (*p* = 0.001). Median ex vivo normothermic heart perfusion time in organ care system was 348.4(132; 955) min. There was significant difference in total out of body time between OCS group 423(67) Vs CS group 210(23) min (*p* = 0.002) Table 2.

**Table 2:** Outcomes data

	OCS (*n* = 25)	CS (*n* = 10)	*P* value
Total ischemic time (minutes)	74.6 ± 13	210 ± 23	< 0.001
OCS perfusion time (minutes)	348.4 (132;955)	NA	NA
Mean total out of body time (minutes)	423 ± 67	210 ± 23	0.002
Warm ischemic time (minutes)	53.4 ± 12.3	60.2 ± 11.5	0.8
MCS after Htx (%)	24	60	0.02
CPB time (minutes)	279 ± 87	256 ± 69.2	0.4
Duration Inotropic support (hours)	103 (47; 465)	236 (153;423)	0.1
ITU stay-days	16 (3;50)	20 (12; 52)	0.3
30-day survival (%)	96	100	0.5

*CPB* cardiopulmonary bypass

**Fig. 1:**
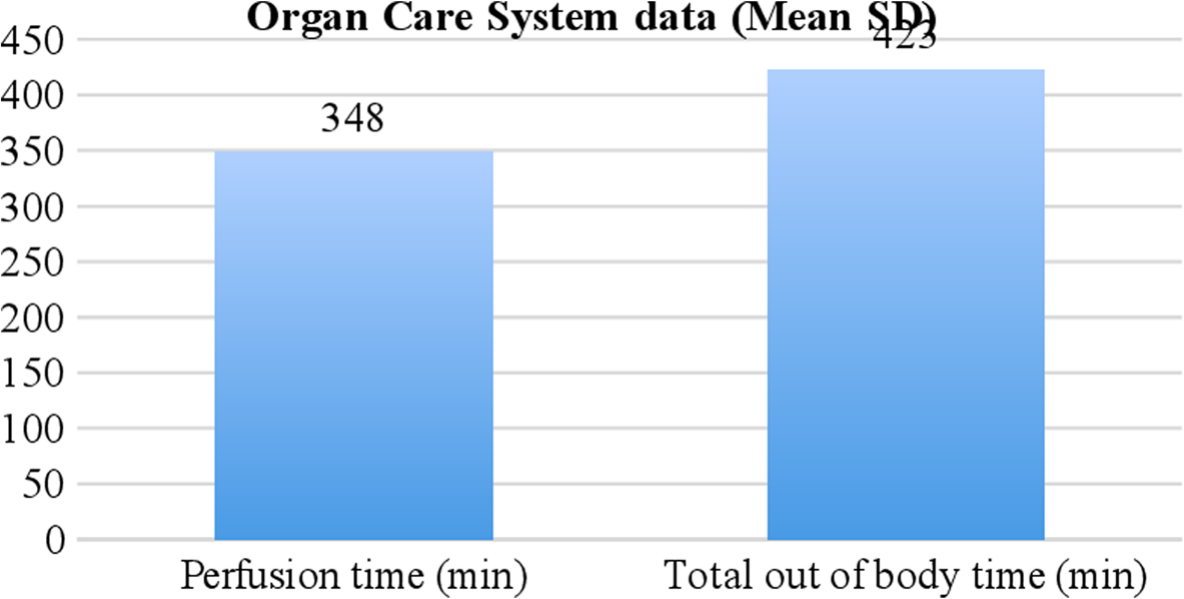
Organ Care System data (Mean SD)

**Fig. 2:**
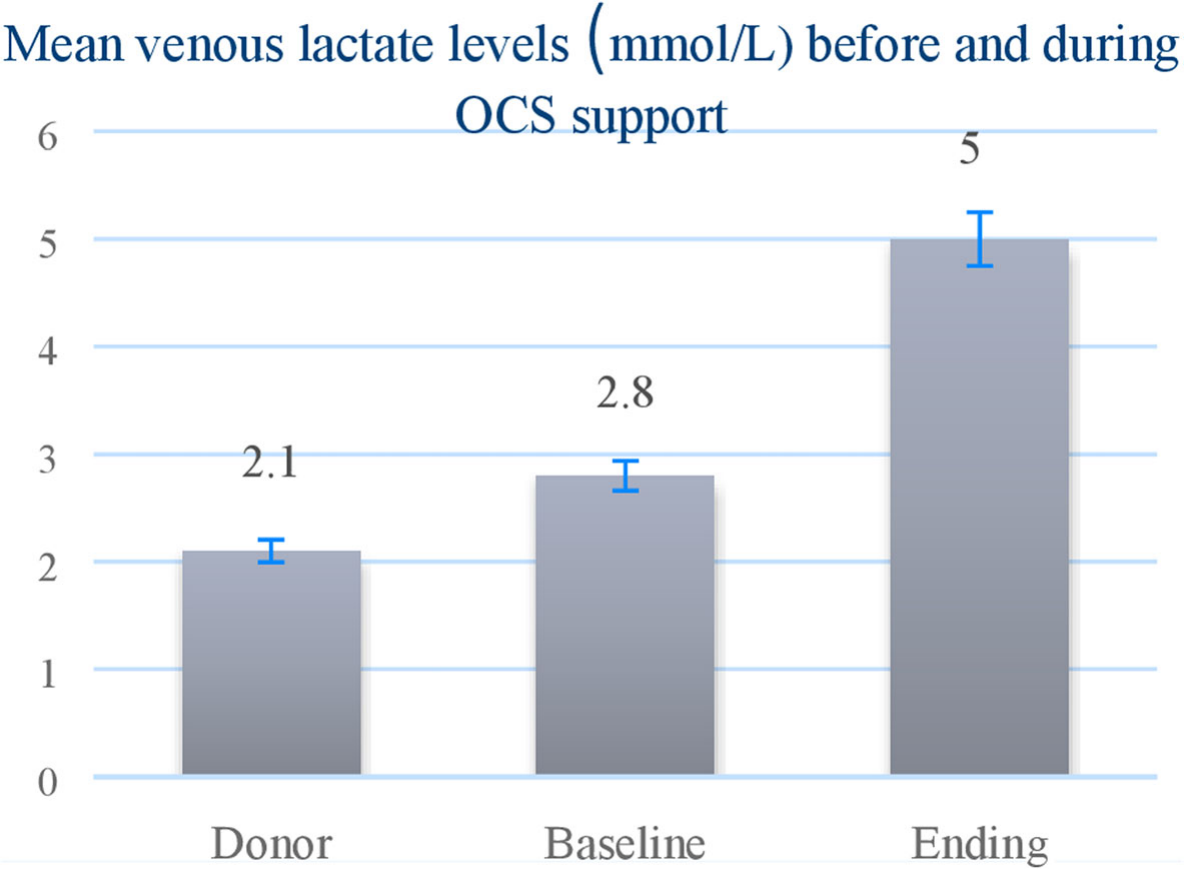
Venous lactate level (mmol/L) before and during OCS support (Mean SD)

In the OCS group, allograft had stable perfusion and biochemical characteristics during ex vivo perfusion (Fig. 1). Mean venous lactate trend during perfusion is normal level (Fig. 2).

### Intraoperative and Postoperative course and survival

The mean warm ischemic time for heart implantation was 53.4(12.3) vs 60.2 (11.5) minutes *p* value 0.8 in OCS and CS group. The allograft total ischemic time was 74.6(13) vs 210(23) minutes *p* value < 0.001. The mean cardiopulmonary bypass time was 279(87) vs 256 (69) minutes *p* value 0.4. Six (24%) patients in OCS (one patient had RV dysfunction, one patient had sepsis, and other four had biventricular dysfunction) and six (60%) in CS group (two patients had sepsis, and other four had biventricular dysfunction) required ECLS support for weaning from cardiopulmonary bypass (*p* = 0.02). In all the cases, the allograft function improved and ECLS could be weaned median after 4 days, except one patient in OCS group who had developed right ventricular dysfunction. The median duration on inotropic support was 103(47; 465) vs 236(153; 423) hours *p* = 0.1, mean level of inotropic support 24 h was dobutamine 7.1(1.6) vs 8.5(1.9) mcg/kg/min *p* value 0.05, milrinone 0.2(0.3) vs 0.25 (0.4) mcg/kg/min *p* value 0.7 in OCS and CS group, respectively. The median ITU stay was 16 days (3; 50) in the OCS group and 20 days (12; 52) in the CS group *p* = 0.3. Inotropic support duration and level was significantly lower in OCS group Table 2.

All patients were alive on the 30th days post implant in CS groups and 96% in OCS group *(p* = *0.5)*.

## Discussion

The preservation of a donor heart before transplantation for a longer period remains an unsolved problem in cardiac surgery. This is very important especially in the countries with low density of population and large distance between the organ procurement to the transplantation site. Organ procurement system might be of utmost importance in this situation. To our knowledge, this is the first clinical report of heart transplantation using the OCS in comparison of standard cold storage for mechanical circulatory support recipients.

The OCS standard protocol allows for extended preservation out of body time of 8 h, for organ procurement, reducing the detrimental effects of cold ischemic storage, improving short-term heart allograft function. In our center, we allow for extended out of body time (more than 8 h as mentioned above) due to geographical distances between donor and recipient hospitals [10].

Based on results from the PROCEED II trial, which implied that a rising lactate level more than 5 mmol/L on the system is a predictor of donor heart abnormality, we made the decision to use the OCS for the assessment of extended criteria donor hearts. Therefore, we compared the results of standard preservation modification with the new approach, using ultrafiltration, levosimendan and blood cardioplegia for conditioning of donor heart during ex vivo perfusion [11]. Ex vivo assessment combined with conditioning would minimize the risk of primary allograft dysfunction and potentially increase the donor pool. The International Society for Heart and Lung Transplantation registry continues to identify LVAD bridging as a risk factor for increased mortality after transplantation. Requirement of mechanical support after transplant indicated as Primary Graft Dysfunction in our analyses. With prolonged ischemic time, the donor heart could now be harvested at more distant areas, expanding the list of potential recipients and increasing the chances of gaining a matching donor heart. This reduced ischemic time is especially beneficial in patients with previous cardiac surgery or in redo transplantation, or in unique situations as HTx after TAH (CARMAT) [12] giving surgeon’s additional time to safely *lyse* all the adhesions and prepare the *cuffs* before arresting the heart in the OCS. Total ischemic time during preservation was statistically significantly longer in CS group in comparison with OCS group. The former caused (significantly) prolonged reperfusion time before disconnection from cardiopulmonary bypass in CS group. Shorter ischemic time, controlled and assessed perfusion in OCS may promise better prognostic outcomes.

OCS Heart allowed safe transplantation of recipients on Mechanical Circulatory Support. Despite preservation mean time was approaching 7 h (maximum 16) enabling allocations otherwise not acceptable, patient and graft conditions were favorable [10]. The ex vivo heart perfusion allows optimization of logistics and meticulous preparation of the recipients with MCS.

Several limitations of this study merit attention. The main was the analysis of a small cohort of patients from a single institution who underwent heart transplantation using the OCS versus cold storage as a method of allograft preservation/assessment. Due to these limitations, the results of this single center should not be generalized to other cardiac surgery population. Full evaluation of a modified preservation for transportation of a donor hearts requires a larger group of patients. Our preliminary findings should be tested in a larger, randomized multicenter trial.

## Conclusions

Preservation of harvested donor hearts to be transported for long distances remains a problem in heart transplantation, especially in patients who are on temporary support on ventricular assist devices. Normothermic ex-vivo preservation of the allograft during transportation with the organ care system might be beneficial for long-time out of body organ preservation in comparison of cold storage especially for recipients on mechanical circulatory support.

## Data Availability

Not applicable.

## Abbreviations

OCS: Organ Care System
MCS: Mechanical Circulatory Support
LVAD: Left ventricular assisted device
IMPACT: Index for Mortality Prediction after Cardiac Transplantation
NICM: Non-ischaemic cardiomyopathy
LVEF: Left ventricular ejection fraction
LVAD: Left ventricular assist device
ECMO: Extracorporeal Membrane Oxygenator
CARMAT: Total artificial heart
